# Rebuilding GeoHealth: Science, Governance, and Resilience After 2025

**DOI:** 10.1029/2026GH001997

**Published:** 2026-10-12

**Authors:** Jennifer R. Bratburd, Gaige H. Kerr, Kishla Askins, Paula R. Buchanan, Bailey Magers, Ray Yeager, Ryan S. D. Calder

**Affiliations:** ^1^ Center for Sustainability and the Global Environment University of Wisconsin‐Madison Madison WI USA; ^2^ Department of Environmental and Occupational Health George Washington University Washington DC USA; ^3^ Polar Pangea Omaha NE USA; ^4^ National Center for Domestic Preparedness (NCDP) Columbia Climate School New York NY USA; ^5^ Department of Civil Engineering Research Foundation of the City University of New York New York NY USA; ^6^ School of Medicine University of Louisville Louisville KY USA; ^7^ Department of Population Health Sciences Virginia Tech Blacksburg VA USA; ^8^ Department of Civil and Environmental Engineering Duke University Durham NC USA

**Keywords:** science policy, GeoHealth, environmental health, science governance, science‐policy interface, scientific infrastructure

## Abstract

Recent U.S. federal policy changes have created disruption and uncertainty for researchers, policymakers, and practitioners in GeoHealth, the interlocking sciences connecting Earth systems to human health and wellbeing. The termination of billions of dollars in research and implementation funding, the dismantling of data systems and regulatory authority, and reductions in scientific workforce capacity have had cascading domestic and global consequences. These changes affect research and public health workflows, health outcomes, and the institutions that create evidence‐based policy. This contributes to growing polarization in trust of science and reduces its policy relevance. In this Article, we assess the implications of these changes for GeoHealth governance and identify strategies to stabilize and rebuild the field. While federal leadership is crucial for scientific funding and policy coordination, the dependence of the research and science‐policy communities on federal resources has created an unacceptable exposure to political volatility and influence. We propose reforms to limit disruption of funded research, expand partnerships with state and local governments, strengthen statutory protections for scientific infrastructure, and develop more reciprocal international collaborations that reduce systemic dependence on U.S. capacity. We also highlight the need to realign academic incentives to support public engagement, actionable research, and evidence‐based policy. These recommendations aim to inform policymakers, research institutions, and funding agencies on the scope of and possible responses to the current crisis in order to preserve the benefits of coordinated federal science while building a more resilient and accountable system of research, policy, and practice.

## Plain Language Summary

Recent U.S. federal policy changes have disrupted GeoHealth, which connects environmental health, Earth systems science, public health, and policy. Cuts to research funding, data systems, regulatory decision‐making, and the scientific workforce are affecting researchers as well as the agencies, practitioners, and communities that make decisions using evidence produced by researchers. This Article explains how these changes weaken the systems that allow science to inform policy. It argues that, while federal leadership remains essential, the current system is too vulnerable to political shifts. When funding, data infrastructure, and expert capacity can be dismantled at a pace that exceeds the time they take to establish, harmful long‐term effects spread across public health, environmental protection, and global scientific collaboration. We propose ways to make GeoHealth more resilient: protecting funded research from sudden disruption, strengthening legal protections for scientific infrastructure, building stronger coordinated partnerships with state and local governments, supporting more equitable international collaborations, and changing academic incentives so researchers are rewarded for public engagement and policy‐relevant work. Lessons learned from the harmful impacts of recent U.S. federal policy shifts highlight the importance of preserving the benefits of coordinated federal science while building a more stable, accountable system for research, policy, and practice.

## Key Points

- Cancellation of grants and loss of key infrastructure and personnel by the U.S. government since 2025 has had global impacts
- The current model for research, practice, and policy engagement creates unacceptable exposure to political volatility
- Federal legal protections, state‐level and international engagement, and greater contact between scientists and communities are needed

## 1 Introduction

The second Trump administration has moved quickly to reshape scientific research and policymaking in the United States and internationally. This has had disproportionate impacts on GeoHealth, the study of interactions among environmental, social, and health systems, and on related fields such as Earth science, environmental health, public health, and climate science that generate foundational knowledge and support public health and policy interventions. Early actions have included the mid‐performance termination or suspension of thousands of active research awards totaling more than $1.3 billion and environmental justice implementation projects totaling more than $28.3 billion as of March 2026 (Delaney, 2026); restructuring or elimination of federal agencies and offices, removing mechanisms scientific consultation and congressional oversight (King, 2025; Marisam, 2025; PBS/WETA, 2025); weakening environmental protections (U.S. EPA, 2026c, 2026d); withdrawing the U.S. from participation in international treaties, scientific bodies, and policy fora (Executive Office of the President, 2025; U.S. HHS, 2026); and diverting scientific investments away from discovery and toward development of artificial intelligence (AI) technologies and commercialization (Nature, 2026; The White House, 2026).

Federal courts and the Congressional Government Accountability Office (GAO) have found that many of these actions violate federal laws including the Administrative Procedures Act and the Impoundment Control Act (SCOTUS, 2025a; U.S. District Court D.S.C., 2026; U.S. GAO, 2025). More recently, Congress has sought to preempt via legislation executive rulemaking efforts that would further disrupt scientific independence by reducing the role of peer review in grantmaking and expand political authority to cancel already awarded grants (OMB, 2026; U.S. Senate Committee on Appropriations, 2026b). These responses are however incomplete because (a) the U.S. Supreme Court has ruled that disputes over canceled grants must be heard by the U.S. Court of Federal Claims, which does not have the authority to order grants to be restored (SCOTUS, 2025a); (b) diverse other standing, cause‐of‐action, jurisdictional, and remedial doctrines (including recent limits to the scope of national injunctions) may complicate legal remedies to unlawful executive actions (SCOTUS, 2025b; U.S. CRS, 2026; U.S. Court of Appeals, D.C. Cir, 2025); and (c), many actions already taken have had irreversible consequences, particularly on individual research trajectories, including workforce attrition, interruption of data collection, loss or destruction of physical infrastructure, impacts on workforce mental health, and early career professionals permanently leaving the field.

These impacts extend beyond science and public health to the legitimacy of democratic governance itself. Scientific institutions provide independent expertise, structure deliberation, and justify decisions in terms that are publicly contestable rather than purely subjective or political (Guston, 2001; Jasanoff, 2010). Legal and policy scholarship has therefore increasingly characterized regulatory science, executive agencies, and expert advisory systems as components of a broader “fourth branch” of government that constrains arbitrary decision‐making and enables accountability (Goodwin & Sinclair, 2021; Livermore & Revesz, 2021). The marginalization of independent sources of expertise and scrutiny is therefore a recurring feature of democratic backsliding (King, 2025; Stockemer, 2025). Indeed, a focus on individual legal challenges and rulemaking efforts may overlook the broader context of how science is likely to be shaped by a government willing and able to use available leverage to marginalize independent institutions (Fernandez, 2026; Roberts Lyer et al., 2022; Várszegi, 2023). Notably, the recent upheaval in federal science emerges from Project 2025, a blueprint for federal governance developed by a coalition of conservative organizations, in which greater control of science is intertwined with a maximalist interpretation of presidential control over the entire federal bureaucracy and the use of federal leverage to exert control over universities, nonprofits, and other institutions of civil society (Lewis, 2026; Piroddi et al., 2026; Resh, 2026).

Disruptions to GeoHealth research and relevant policies in the United States have had global effects. The U.S. federal government accounts for ∼27% of worldwide research and development (R&D) expenditures (National Science Board, 2022), ∼31% of worldwide public and philanthropic health research funding (Røttingen et al., 2013), and about half of worldwide health research funding between 2007 and 2023 (Impact Global Health, 2025). U.S. federal agencies provide a disproportionate share of the global scientific infrastructure (e.g., satellites, databases, human cohort studies, etc.) on which research programs around the world depend. As the world's second largest greenhouse gas emitter, its withdrawal from the Paris Climate Agreement severely limit the effectiveness of the overall framework (U.S. CRS, 2025). U.S. politicization of science has led international organizations including the IEA and the World Bank to deprioritize the climate crisis (Giordano, 2026; Schonhardt & Colman, 2025).

In this Article, we document the losses to GeoHealth to date, identify strategies to resist further erosion, and propose pathways to rebuild the field. We argue that a model resilient to such shocks will require institutions less vulnerable to federal and/or short‐term political swings; this includes stronger statutory foundations for federal science, expanded collaborations with local, state, and international actors, and broader public engagement. Overall, the present disruption exposes opportunities to both correct structural vulnerabilities and catalyze a more resilient, engaged and accountable scientific enterprise that is better able to leverage state and local policy authority, with implications for environmental health and science‐based governance more broadly.

## 2 Losses and Vulnerabilities

Since 2025, U.S. society has lost a substantial fraction of the interlocking research infrastructure, implementation programs, data systems, regulatory authority, and institutional resources that underpin GeoHealth research, policy, and public health action (Table 1). These losses expose the fragility of the assumptions on which U.S. Earth sciences and environmental health infrastructure is built, namely, stable, bipartisan commitments to scientific leadership and independence backstopped by broad public support. These losses are felt most acutely in communities that are already overburdened and under‐resourced, exacerbating already significant disparities that are both unjust and a contributor to further social instability (De Juan & Wegner, 2019; Hunnicutt & Henderson, 2023; Walther & Chou, 2023).

**Table 1:** Key GeoHealth Programs, Policies, and Assets Either Already Lost (Red) or at Risk (Yellow) as of Spring 2026

Program, policy, or asset	Description	Status	Impact	References
Environmental health research grants (EPA, NIH, NSF)	Federally funded research programs that generate the scientific evidence base for environmental health policy, including studies of toxic exposures, air pollution, climate‐related hazards, and environmental determinants of disease. These projects are typically conducted by universities in collaboration with community‐based partners, state agencies, and other non‐governmental organizations	$1.3 billion including virtually all EPA STAR awards terminated; numerous lawsuits pending including APHA et al. v. NIH, AAUP v. Trump, Harvard v. HHS et al., etc	Immediate loss of ongoing environmental health research and training pipelines; interruption of longitudinal data sets and early‐career researcher support; billions of dollars in economic activity in award institutions' communities; unknown number of graduate students and trainees now unemployed	AAUP (2025), APHA (2025), Delaney (2026), and Harvard (2025)
Federal scientific workforce (EPA, NOAA, NASA, etc.)	Federal scientists and technical staff responsible for environmental monitoring, risk assessment, regulatory analysis, and scientific advisory functions	Substantial attrition in 2025, with >10,000 doctoral‐level scientists leaving federal service; hiring constrained	Loss of technical capacity across monitoring, modeling, and regulatory science; reduced ability to maintain observational systems, conduct risk assessments, and develop environmental health policy. Workforce losses diminish institutional memory, creating long‐term reductions in state capacity	Mervis (2026)
NOAA Billion‐Dollar Weather and Climate Disasters database	Data set maintained by NOAA that cataloged U.S. weather and climate disasters causing ≥$1 billion in losses since 1980. The database integrated information from diverse sources to produce standardized estimates of disaster frequency and economic losses across hazard types	Retired in 2025 with no updates beyond 2024. Archived historical data remain accessible, but new events are no longer incorporated. Subsequent tracking has been partially continued by non‐governmental organizations using the original NOAA methodology	Loss of the federal benchmark for the economic and societal costs of extreme weather widely used by scientists, insurers, investors, emergency managers, and policymakers. From 1980 to 2024 the NOAA database documented $2.8 trillion in damages, rising sharply in the period 2020–2024. The database underpinned insurance risk modeling, infrastructure planning, disaster preparedness, and climate‐risk assessment	Climate Central (2025), NCEI (2025), and Popilek (2025)
EPA EJScreen	Federal geospatial screening tool integrating environmental exposure indicators (e.g., air pollution, proximity to hazardous waste sites, traffic, lead paint risk) with demographic vulnerability indicators (e.g., income, race, linguistic isolation) to identify communities experiencing disproportionate environmental burdens	Removed from EPA website in 2025; historical versions remain archived but are no longer updated	Removal eliminates the federal government's primary standardized framework for identifying environmental justice communities. EJScreen informed federal permitting, regulatory analyses, community advocacy, and academic research. Because relatively few states maintain comparable screening tools, its removal also eliminates the only consistent nationwide data set for identifying cumulative environmental health burdens	EDGI Team (2025) and U.S. EPA (2024)
EPA Value of a Statistical Life (VSL)	Economic valuation used by the U.S. Environmental Protection Agency and other federal agencies to quantify the benefits of regulations that reduce mortality risk. The VSL is a central parameter in cost‐benefit analyses for air pollution standards, chemical regulations, transportation safety, and other public health protections	In 2026 the administration announced that EPA would no longer apply the Value of a Statistical Life when evaluating regulations affecting stationary pollution sources, substantially narrowing the use of mortality risk valuation in regulatory impact analysis	Removing VSL from regulatory analyses for major pollution sources reduces the quantified benefits of environmental health protections, potentially weakening justification for rules controlling industrial emissions, hazardous air pollutants, and climate‐related health risks. Because mortality risk reductions often account for the majority of monetized benefits in air pollution regulations, limiting VSL use could significantly alter cost–benefit outcomes for environmental rules affecting millions of Americans	Robinson et al. (2019), Sarofim (2026), and U.S. EPA (2026c)
NOAA GeoXO mission scaled back (Atmospheric Composition Instrument removed)	Planned next‐generation U.S. geostationary satellite system intended to succeed the GOES‐R series beginning in the early 2030s. GeoXO would provide continuous observations of weather, atmospheric composition, and ocean conditions, including high‐frequency measurements of air pollutants and wildfire smoke using the Atmospheric Composition Instrument (ACX)	NOAA removed the Atmospheric Composition (ACX) instrument and other environmental sensors from the baseline GeoXO mission architecture during mission restructuring in 2025	Loss of planned hourly geostationary monitoring of air pollution and atmospheric chemistry across North America, reducing future satellite capacity for air‐quality forecasting, wildfire smoke tracking, and environmental health exposure assessment. These observations were expected to support pollution forecasting and public health advisories for hundreds of millions of people across the United States	Dance (2025) and Fernholz (2025)
U.S. Embassy Air Quality Monitoring Network	Global network of air quality monitors operated by the U.S. Department of State providing real‐time PM2.5 data in cities worldwide, especially where no reliable local data exist	Monitoring halted in 2025 and public dissemination discontinued	Loss of a key global public health data source used by researchers, local political actors, and the public. Research suggests that this data contributed to large reductions in air‐pollution‐related mortality	Friedman and Plumer (2025)
Greenhouse Gas Endangerment Finding	EPA determination under §202(a) of the Clean Air Act that greenhouse gases endanger public health and welfare following the Supreme Court ruling in Massachusetts v. EPA (2007). The finding provides the legal basis for federal regulation of greenhouse gas emissions from vehicles, power plants, and other sources	EPA finalized rescission of the Endangerment Finding in 2026 as part of a rulemaking eliminating federal greenhouse gas standards. Multiple lawsuits have been filed by states, environmental organizations, and others challenging the repeal and regulatory rollback	Repeal removes the foundation for federal greenhouse gas regulation under the Clean Air Act, shifting responsibility to states and weakening national coordination of climate policy. Modeling suggests the rollback could eliminate a large share of projected U.S. emissions reductions while increasing risks associated with extreme heat, air pollution, wildfire smoke, flooding, and other climate‐related health hazards	APHA (2026), U.S. EPA (2026d), and Wooten et al. (2026)
NASA Research Opportunities in Space and Earth Sciences (ROSES) environmental and climate justice research theme	NASA's ROSES‐2022 A.28 Interdisciplinary Research in Earth Science program included an “Environmental and Climate Justice Using Earth Observations” theme supporting research that used Earth observations to examine disproportionate environmental, health, and climate‐change impacts	Not solicited in 2024; later disrupted by implementation of executive orders targeting DEI activities and other disruptions following pauses to federal assistance	Loss of interdisciplinary GeoHealth research capacity connecting satellite observations, climate modeling, and community health applications. Terminations disrupted partnerships with frontline communities and reduced decision‐support capacity for climate adaptation, hazard preparedness, and environmental justice planning	
EPA Avoided Emissions and geneRation Tool (AVERT) and EPA CO‐Benefits Risk Assessment (COBRA) Tool	Screening tools used to estimate emissions, air quality, health, and economic benefits and impacts of alternative energy, climate, and air pollution policies. AVERT estimates emissions changes from alternative pathways in the energy sector, while COBRA estimates health and other impacts	Publicly available (desktop and web‐based) as of summer 2026, but vulnerable to loss given other agency actions around climate	Loss or degradation would reduce capacity of governments and other actors to estimate health co‐benefits of emissions reductions, compare benefits against program costs, and map air‐quality and health impacts of interventions in the energy sector	U.S. EPA (2026a, 2026b)
National Climate Assessment	Congressionally mandated scientific assessment synthesizing the state of climate science and observed and projected climate impacts across sectors in the United States, including public health, infrastructure, water resources, agriculture, and ecosystems. It has served as a foundational reference for climate adaptation planning, risk assessment, and policy analysis across sectors	Work on the Sixth National Climate Assessment halted in 2025 after dismissal of nearly 400 contributing scientists and disruption of the U.S. Global Change Research Program; the congressionally mandated report scheduled for release around 2027–2028 is now uncertain. AGU and AMS plan to release a stand‐in report	Disruption or delay eliminates the federal government's primary authoritative synthesis of climate risks used by federal and state agencies, infrastructure planners, and public health officials	AGU & AMS (2025), Plumer and Dzombak (2025)
Mauna Loa Observatory atmospheric CO_2_ monitoring	Continuous atmospheric monitoring program operated by NOAA and partners at Mauna Loa Observatory, Hawaii. Since 1958 the site has produced the longest uninterrupted record of atmospheric carbon dioxide concentrations, known as the Keeling Curve, widely used to track global greenhouse gas trends	Federal funding reductions and operational restructuring announced in 2025 threaten continued operation of the observatory and associated long‐term atmospheric monitoring activities.	Potential disruption of the longest continuous atmospheric CO_2_ record, a foundational data set for climate science, Earth system modeling, and global emissions assessment. The Keeling Curve underpins international climate assessments (including IPCC reports), carbon cycle research, and policy evaluation of emissions trends. Interruptions in long‐term monitoring would reduce the scientific value of a record spanning more than 65 years	Gupta et al. (2025) and Keeling et al. (1976)
National Center for Atmospheric Research (NCAR)	Federally funded research center that develops major climate and weather modeling systems (including the Community Earth System Model) and operates national atmospheric observing and supercomputing infrastructure used by researchers, forecasters, and environmental health scientists worldwide	Policy proposals and agency restructuring plans since 2025 have raised the possibility of redistributing, privatizing, or reducing federal support for NCAR functions, including weather modeling, research facilities, and atmospheric observing programs	Potential disruption to core modeling and observational infrastructure underpinning U.S. climate and weather science, widely used in IPCC assessments, forecasting, wildfire and air‐quality modeling, and environmental health research. NCAR data sets also support climate‐risk assessment by insurers, infrastructure planners, and water managers. Reduced federal support could weaken U.S. leadership in climate modeling and limit access to shared scientific infrastructure used by hundreds of universities and research institutions	Richter (2025) and UCAR (2025)

Canceled or lost
Threatened

### 2.1 Research Infrastructure and Implementation Systems

Since 2025, the second Trump administration has reshaped the U.S. GeoHealth field by terminating approximately $29.6 billion in Congressionally appropriated funding administered by the National Science Foundation (NSF), the National Institutes of Health (NIH), the Environmental Protection Agency (EPA), and other U.S. federal agencies, affecting universities, state agencies, nonprofits, and Tribal organizations. This spans both research activities (≥$1.3 billion) and non‐research activities (≥$ 28.3 billion) to reduce hazards such as lead exposure, extreme heat, and other climate risks. The community‐based implementation grants classified as “non‐research” directly reduce burden of disease and are vastly cost effective, with programs such as lead abatement returning $17–$221 in societal benefits per dollar invested through reduced healthcare costs, increased lifetime earnings, and lower public expenditures (Gould, 2009). Many of these awards were funded through the Environmental and Climate Justice Program established by the Inflation Reduction Act (∼$3 billion), which had been a highly significant source of resources for states with no comparable state‐level analogs. Grants classified as “non‐research” are primarily intended to support community‐based implementation but may also include funding for research activities.

The elimination of the EPA Science to Achieve Results (STAR) program removes a primary federal funding stream for GeoHealth and other interdisciplinary environmental health sciences. These areas often fall between the missions of the NIH and the NSF, leaving few alternative sources of support (Calder & Schartup, 2023). This is in addition to the terminated research identified as relevant to GeoHealth funded by the NIH ($ 4.0 million) and NSF ($ 28.4 million). Figure 1 illustrates the spatial distribution of recent cuts and shows that they affect both centralized research capacity in major academic hubs and community‐level programs in less‐resourced states. A substantial share of canceled commitments for non‐research and implementation activities is distributed across interior and Southern states, which disproportionately lack state‐level capacity to mitigate environmental hazards and/or inequities (explaining why they received the grants in the first place). Because the data set underlying Figure 1 combines researcher‐submitted information with government records, it may not capture all terminated awards, particularly those that have not yet been reported or identified in available sources. Additionally, estimates of remaining funds are subject to limitations in federal spending data and, for NIH awards, assumptions of constant spending over the grant period.

**Figure 1:**
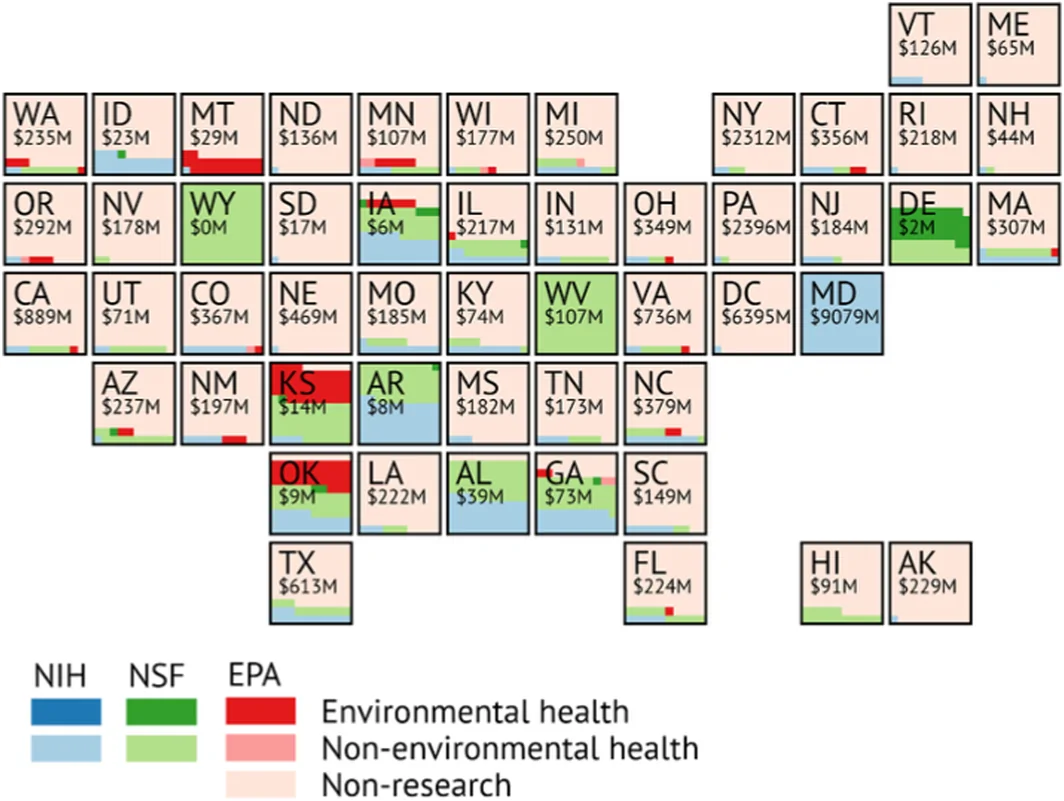
State‐level federal grant terminations by agency and relevance to environmental health from Grant Witness. Inset text indicates the total amount of remaining funding from terminated grants in millions of USD. Colors represent the proportion of this the terminated funding from the National Institutes of Health (NIH), the National Science Foundation (NSF), and Environmental Protection Agency (EPA), and the tint of the colors denotes grants' potential relevance to environmental health and/or research (see Table S1 for environmental health and research inclusion criteria).

### 2.2 Data Infrastructure and Regulatory Authority

Widely used federal data portals central to GeoHealth, as well as severe weather prediction and response tools, have been removed or disrupted. Examples include EPA's EJScreen mapping tool, the Greenhouse Gas Emissions Inventory, the NOAA Billion‐Dollar Weather and Climate Disasters database, and the National Climate Assessment (NCEI, 2025; Volcovici, 2025; Woods, 2025). The restructuring of EPA's Office of Research and Development has further reduced federal capacity to conduct the analyses that underpin environmental standards (Daly, 2025). Because fewer than half of U.S. states maintain comparable tools (Marvin & Maye, 2025), these losses disproportionately affect jurisdictions with limited independent capacity to identify and respond to environmental hazards.

More fundamentally, the February 2026 rescission of the 2009 Greenhouse Gas Endangerment Finding eliminated EPA authority to regulate greenhouse gases under the Clean Air Act, removing the legal basis for approximately a dozen nationwide emission standards (U.S. EPA, 2026d). Modeling suggests this repeal could impose roughly $800 billion in net social costs by 2050 (Linn & McCormack, 2026). Concurrent changes to benefit‐cost methodology that exclude valuation of mortality risk further undermine the quantitative basis for future environmental health interventions (U.S. EPA, 2026c), marking a departure from the bipartisan role of cost‐benefit analysis as a shared framework for regulatory decision‐making (Livermore & Revesz, 2021).

The loss of critical infrastructure and talent are likely to undermine the Administration's own stated priorities. Biosecurity and economic competitiveness feature prominently in federal research guidance for FY2027 (Vought & Kratsios, 2025), yet reductions in disease surveillance capacity and global health funding create precisely the conditions under which emerging infectious threats go undetected. Meanwhile, cuts to the U.S. research enterprise risk ceding ground to China and Europe in strategically important technologies (The Economist, 2025; Tollefson et al., 2026).

### 2.3 Scientific Workforce and International Capacity

More than 10,000 doctoral‐level scientists left federal employment in 2025, with departures in some agencies outpacing new hires by roughly 11 to 1 (Mervis, 2026). Bureaucratic expertise is a core component of regulatory competence (Dahlström & Lapuente, 2017); GeoHealth and other environmental health sciences are especially sensitive to these losses because they depend on sustained observational systems, longitudinal cohorts, and specialized risk assessment capacity that require long time horizons to rebuild. Prolonged funding instability has compounded this through hiring freezes, suspended graduate training programs, and rescinded offers for early‐career scientists (Woolf et al., 2025), while Europe and China have expanded recruitment efforts during this period of U.S. contraction (The Economist, 2025; Tollefson et al., 2026).

The effects extend globally. The termination of the U.S. embassy air quality monitoring network eliminated the only reliable air pollution measurements in several countries and removed a program credited with catalyzing policy reforms and contributing to large reductions in air pollution‐related mortality worldwide (Friedman & Plumer, 2025; Jha & Nauze, 2022). U.S. withdrawals from the Paris Agreement, the IPCC, and the WHO further reduce the international scientific and governance infrastructure on which domestic and global environmental health depends (Castelvecchi & Masood, 2026; Executive Office of the President, 2025; U.S. HHS, 2026). Reductions in U.S. global health funding have raised concerns about the continuity of disease surveillance systems, with modeling suggesting large downstream effects on mortality in low‐income countries (Cavalcanti et al., 2025).

Beyond funding cuts, federal uncertainty and policies cause major impacts on research and scientists. For example, even without the realization of proposed large cuts in health research funding to the National Institutes of Health, a ∼10% sample of the 989 U.S.‐based members of the International Society for Environmental Epidemiology reported severe impacts: 97% of respondents reported adverse impacts on their career stability; 95% on their ability to conduct research; and 99% on their mental health. Over 70% of respondents reported increased anxiety over job security, burnout/exhaustion, difficulty concentrating on research, and disillusionment about academic work. Impacts on career stability were especially pronounced among early career researchers (ISEE, 2026). In a separate survey of NIH grantees, 43% of respondents have canceled planned research projects, with 66% encouraging students to consider career paths outside of academia and 53% outside of the U.S. (MPG, 2026). With much of the future environmental health workforce now unable or unwilling to pursue careers in the field, the culmination of such impacts will have lasting harms on the research and public health workforce over decades to come.

### 2.4 Presumption of Scientific Independence

Environmental and public health governance depends on scientific information generated independently of political influence. Public confidence in scientists remains comparatively high overall but is increasingly polarized: in January 2026, Pew found that 89% of Democrats expressed confidence in scientists acting in the public interest, compared with 58% of Republicans, a gap that has widened markedly since 2018 (Milkoreit & Smith, 2025). Research attributes the decline in trust more strongly to partisan political signaling and media ecosystems than to the content of research itself (Druckman & McGrath, 2019; Gauchat, 2012; Rekker, 2021). Actions that publicly discredit agencies, remove data sets, or portray scientific conclusions as politically contingent accelerate this erosion, further undermining the institutional norm that has historically enabled scientific evidence to serve as a shared basis for environmental health policy. At the same time, this also indicates the need for more scientific engagement, public‐oriented communications, and transparency in the systems that translate evidence into sound policy.

## 3 Holding the Line

### 3.1 Legal and Political Resistance

Courts have in several cases ordered agencies to restore or continue disbursement of research funds, affirming that the executive branch cannot unilaterally withhold Congressionally appropriated funds. However, many rulings apply only to plaintiffs in specific litigation, leaving institutions in states whose attorneys general have not joined legal challenges without equivalent protection (NACUBO, 2025). Furthermore, the Supreme Court has held that contractual disputes between grantees and the federal government must be adjudicated in the Court of Federal Claims, which is charged to award monetary penalties but may not be able to order the government to resume terminated contracts (SCOTUS, 2025a). Nevertheless, sustained legal pressure requires broad coalitions of universities, states, nonprofits, and Tribal organizations willing to join or initiate litigation; recent rulings suggest that arguments viewpoint discrimination claims may be more likely to support injunctive relief in district courts than claims based on breach of contract, which are likely to be channeled to the Court of Federal Claims (U.S. Court of Appeals, 9th Cir, 2026). Congress can use appropriations and other legislation to give legal force to norms of scientific governance that previously depended on agency practice, such as the primacy of expert peer review in federal grantmaking decisions (U.S. Senate Committee on Appropriations, 2026b). Indeed, Congress has maintained strong support for overall science funding, for example, rejecting requests to cut NSF and NASA funding by 57% and 47%, respectively (U.S. Senate Committee on Appropriations, 2026a).

Actions of federal executive agencies may also justify increased action at the state level, particularly where there is already state capacity; for example, the rescission of the Endangerment Finding and the removal of federal climate standards may increase interest in expanding or strengthening: programs such as the Regional Greenhouse Gas Initiative and California's AB32 already operated alongside federal frameworks, and their expansion (along with analogous initiatives in other states) may partially offset the loss of federal regulatory coverage, though at the cost of increased interstate policy divergence.

### 3.2 Data and Knowledge Preservation

Federal agencies have removed from their websites or discontinued updates to several widely used data sets and have canceled congressionally mandated scientific assessments. Some of these gaps have been filled by independent organizations: EJScreen has been archived on the Harvard Dataverse (Ansolabehere & Carlson Sirvent León, 2024), the Environmental Defense Fund obtained the Greenhouse Gas Emissions Inventory via a Freedom of Information Act request (EDF, 2025), and researchers are independently completing the National Nature Assessment (retitled “The Nature Record”) after its cancellation (Einhorn, 2026). These efforts are however at best incomplete, as federal, state, and local policymakers are in many cases legally required to rely on authoritative federal sources and independent alternatives or archives may not carry equivalent legal standing. Sustained investment in independent data infrastructure and advocacy for the legal standing of non‐federal data sources may thus be useful complements to ongoing archiving and research substitution efforts.

### 3.3 Protecting At‐Risk Infrastructure

Major components of the U.S. GeoHealth research system are under threat. The National Center for Atmospheric Research (NCAR) runs atmospheric observation and climate modeling programs including the Community Earth System Model used in international climate assessments, extreme weather forecasting, and wildfire smoke transport modeling. In January 2026, NSF issued a request for proposals to redistribute or privatize core NCAR functions (NSF, 2026; UCAR, 2025). This would be highly disruptive to the integrated modeling systems, supercomputing platforms, and collaborative research networks in which universities, public health agencies, and other users are enmeshed. Similarly, NOAA and NASA remote sensing systems provide continuous measurements of atmospheric composition, land surface temperature, wildfire smoke, and ocean conditions used in air quality exposure assessment, extreme heat monitoring, and vector‐borne disease risk monitoring (Freedman, 2026; NASA, 2024). Proposed reductions in planned instrumentation on future satellites, including the GeoXO mission, and delays in replacement missions threaten long‐term observational continuity.

## 4 Rebuilding GeoHealth

The present crisis exposes structural vulnerabilities that predate the current administration. In the wake of the current disruption, there is an opportunity to rebuild GeoHealth systems in a way that is more effective and resilient. This includes being more engaged with different levels of government, more deeply embedded in the communities it serves, entails more visible decision‐making and societal value, and is less institutionally dependent on a single funding stream whose bipartisan durability is in doubt.

### 4.1 Protecting Federal Science Leadership and Coordination

Federal leadership of science has delivered substantial benefits by enabling coordination across states in settings such as disease outbreak prevention and natural hazard response, and the maintenance of a shared scientific workforce. A federal approach has delivered economies of scale that support nationally complete and consistent data sets, monitoring systems, and regulatory programs under federal law. These arrangements balance an efficient and democratically accountable mechanism for producing equitable health outcomes at scale; therefore, preserving and strengthening the federal‐led system should be our first priority. Yet, as described above, these programs depend on institutional stability that has ultimately not been undergirded by the force of law or by sufficiently strong protections for key programs and personnel. Long‐term GeoHealth resources including monitoring networks, cohort studies, and community partnerships cannot function under the possibility of sudden policy reversal or mid‐performance termination of funding. To preserve the benefits of federal leadership while reducing vulnerability to political disruption, key elements of the GeoHealth enterprise should be more firmly embedded in statute, including core monitoring systems, data infrastructure, and research programs. Additional safeguards could include multi‐year funding commitments insulated from annual appropriations cycles, clearer limits on the discretionary termination of grants absent demonstrated misconduct or noncompliance, and reinforcement of independent scientific advisory processes.

Mechanisms for international collaboration such as NSF partnerships with the Royal Society (United Kingdom) and the Natural Sciences and Engineering Research Council of Canada demonstrate the feasibility of cross‐national research; yet, they remain limited in scale, unavailable in many fields of study (including most parts of GeoHealth), and are constrained by funding rules that prioritize domestic spending. This leaves them poorly suited to developing sustained, shared infrastructure for continental or global environmental risks. Expanding these arrangements so as to promote development of shared monitoring systems and other infrastructure could reduce systemic vulnerability to disruptions in any single country while also addressing longstanding concerns about extractive or “helicopter” research practices. Together, these reforms would retain the scale and coordination advantages of federal leadership while increasing the durability and credibility of GeoHealth governance.

### 4.2 Engaging States as Research Partners

At the same time, the current crisis highlights opportunities to reinforce the research enterprise through partnerships between universities and other actors, notably the state governments by which public universities are controlled and partially operated. In the U.S., many highly consequential environmental health policy levers such as air and water permitting, land use, energy infrastructure, disaster and emergency management are within the jurisdiction of states, tribes, and municipalities. Yet, the postwar expansion of federal research funding progressively oriented university research agendas (including those of state institutions) around federal priorities, leaving state governments relatively peripheral as research partners and dependent on federal agencies for scientific personnel, data systems, and decision support (Geiger, 2009; Pew Charitable Trusts, 2019). Research calibrated to federal funding priorities is therefore often poorly matched to the decision contexts where much GeoHealth and broader environmental health policy is actually made (Cash et al., 2003; Guston, 2001).

In response to recent federal retrenchment, Massachusetts and California have begun to experiment with measures to stabilize research capacity (Chen, 2025; Medsger, 2025). Massachusetts's recent law however represents only a small fraction of prior federal investment and cannot realistically substitute for it, and in most states, fiscal constraints make direct replacement infeasible. The combined state‐level tax base is roughly ¼ of the federal tax base, and federal transfers already account for between 20% and 40% of state budgets (Thiess et al., 2025; U.S. CBO, 2024; USAFacts, 2026). Greater expectations of state leadership in science funding would be difficult to satisfy even in the most well‐resourced states under current fiscal arrangements; federal retrenchment would disproportionately affect poorer states, where it would reduce opportunity and diminish a significant source of economic growth, employment, and social development in areas most in need of federal investment in general (Hausman, 2022; Lanahan et al., 2016; Levine & McKnight, 2025).

There may however be mechanisms for state governments to make better use of resources they are already paying for, including faculty and staff salaries at state institutes of higher education. States governments, individually or collectively, may explore mechanisms to partially buy out faculty time from time otherwise expected to be committed to federally funded research to support decision support, regulatory analysis, and applied environmental work within state agencies. Increased cooperation between state research universities and state agencies provides another potential mechanism for university faculty to demonstrate their value to their institutions and to society, in a setting where canceled grants and reduced opportunities at the federal level are complicating the promotion and tenure process. The Extension system provides one model for embedding scientific expertise in local decision‐making, although these have historically relied on federal coordination; analogous state‐led programs could strengthen institutional linkages between universities and policy. An existing array of interstate agreements such as the Regional Greenhouse Gas Initiative and watershed management compacts shows that states can coordinate on environmental policy beyond federal institutions; yet, they rarely pool scientific and analytical capacity. If states take on greater responsibility and build in‐house capacity to monitor and regulate their environments, economies of scale could be realized by interstate coordination along the lines of existing agreements. Block grant models may offer an additional pathway, providing states with flexible federal funding tied to broad environmental health objectives while allowing adaptation to local priorities and conditions and with visible local economic benefits.

Here, too, states have great variability in capacity; increased reliance on such state‐led arrangements may increase the risk that less well‐resourced states become less able to monitor and control environmental hazards beyond or even up to federal minimum standards (Oza, 2026). Overall, however, the current moment may reveal opportunities for more effective use of state resources to shape research agendas and increase the use of expert knowledge and analysis in state‐level decision‐making, and increase resilience to federal funding volatility.

### 4.3 Broadening the Range of Constituencies Invested in Environmental Health Science

Government programs and activities with widespread social awareness and support (e.g., Social Security, Medicare) have historically been much more politically difficult to defund or cut (Jacobs & Mettler, 2018; Pierson, 1994). Although clean air, safe drinking water, and resilient ecosystems provide health and economic benefits to all Americans, the research and policy programs responsible for these benefits have become invisible to too many (U.S. EPA, 2011). In this way, GeoHealth and the environmental sciences may be the victim of their own success since the 1970s, when smog, contaminated rivers, and lead poisoning first galvanized public support for the field (Downs, 1972). Building and sustaining a durable constituency will thus require the field to make its contributions more visible in the context of its own success; science education and outreach is therefore critical to sustain public support for scientific research and institutions (Yacoubian, 2018). Strengthening the nexus between scientific research and local priorities and needs, while also modernizing scientific communications channels, may be another way to increase public awareness of and support for the research enterprise.

### 4.4 Reforming Academic Incentives

Beyond efforts to reinforce the federal‐led status quo, all efforts depend on reimagining academic incentives, including criteria and guidelines for promotion and tenure. At present, both public and private universities prioritize success at securing competitive federal grants, the solicitation and performance of, and the reporting for which, have represented increasing time commitments for tenured and tenure‐track faculty (Alberts et al., 2014; Stephan, 2015). This system diverts substantial effort away from research itself and leaves the researchers best positioned to do sustained policy translation and public engagement with little capacity or institutional incentive to do this work. Yet, scientific expertise sustains democratic legitimacy only by actively entering public deliberation (Habermas, 1984; Kitcher, 2001); disincentivized public engagement of scientists may thus play a role in the fragile public appreciation and support for the scientific apparatus we are currently experiencing. Beyond funding reforms, promotion and tenure guidelines may be reformed to explicitly value public engagement, science communication, and partnership with state and local agencies, not as peripheral service, but as fundamental contributions that make the entire scientific enterprise politically sustainable. Likewise, given the substantial impacts on early‐career researchers, supporting opportunities for students and trainees in GeoHealth disciplines, as well as to learn science communication skills and opportunities to translate their research to policy would be valuable.

## 5 Conclusion

Beyond the upheaval in GeoHealth and allied fields, the pace of change since 2025 serves as a warning about the fragility of the public institutions that sustain science and public health more generally. Policymakers, funders, and academic leaders must act now to protect core scientific infrastructure, reduce political vulnerability, and create a more resilient system of research, practice, and policy. The 2025–2026 period has demonstrated that a scientific apparatus built primarily on shared norms, rather than legally binding safeguards are inadequate to provide the stability needed for scientific research absent political and social consensus on the benefits of scientific independence and autonomy. A more resilient model must therefore pair the scale and coordination advantages of federal leadership with stronger statutory protections, deeper engagement with state and local partners, and a broader base of public investment in and awareness of scientific work.

## Conflict of Interest

Several of the authors have been funded by grant sources described in this paper.

## Supporting information

Table S1

## Data Availability

Filters applied to GrantWitness data (publicly available via www.grantwitness.org) to produce Figure 1 are included in Table S1. The analysis was conducted and figures generated using Python 3.10 (Python Software Foundation, 2021; https://www.python.org/downloads/release/python‐3100/).
