# Supplementary material for: Rebuilding GeoHealth: Science, Governance, and Resilience After 2025
Source: Geohealth. 2026 Oct 12;10(10):e2026GH001997. doi: 10.1029/2026GH001997 (PMC13655975; doi:10.1029/2026GH001997)
Supplement: Supplementary file 1 [file GH2-10-e2026GH001997-s001.pdf]

Supporting Information

Rebuilding GeoHealth: Science, Governance, and Resilience after 2025

Jennifer Bratburd[a], Gaige H. Kerr[b], Kishla Askins[c], Paula R. Buchanan[d], Bailey Magers[e], Ray Yeager[f] & Ryan S.D. Calder[g,h]*

[a] Center for Sustainability and the Global Environment, University of Wisconsin-Madison, Madison, WI, USA
[b] Department of Environmental and Occupational Health, George Washington University, Washington, DC, USA
[c] Polar Pangea, Omaha, NE, USA
[d] National Center for Domestic Preparedness (NCDP), Columbia Climate School, New York, NY, USA
[e] Department of Civil Engineering, Research Foundation of the City University of New York, New York, NY, USA
[f] School of Medicine, University of Louisville, Louisville, KY, USA
[g] Department of Population Health Sciences, Virginia Tech, Blacksburg, VA, USA
[h] Department of Civil and Environmental Engineering, Duke University, Durham, NC, USA

* Contact: rsdc@vt.edu; phone: (540) 231-2430; fax: (540) 231-7007

Table S1. Filter fields and included values used to designate terminated grants on Grant Witness (https://grant-witness.us/) as "environmental health," "non-environmental health," and "non-research" in Figure 1.

EPA filter field	Designation	Values
cfda_title	Environmental health	Research Development Monitoring Public Education Outreach Training Demonstrations and Studies, Science to Achieve Results Star Research Program, Surveys Studies Investigations Training and Special Purpose Activities Relating to Environmental Justice
	Non-environmental health	Office of Research and Development Consolidated Research Training Fellowships, P 3 Award National Student Design Competition for Sustainability, Reducing Embodied Greenhouse Gas Emissions for Construction Materials and Products, Surveys Studies Investigations and Special Purpose Grants Within the Office of Research and Development, 'Alternative or Innovative Treatment Technology Research Demonstration Training and Hazardous Substance Research Grants
	Non-research	Environmental Finance Center Grants, Environmental Justice Collaborative Problem Solving Cooperative Agreement Program, Environmental Justice Government to Government Ejg 2 g Program, Environmental Justice Small Grant Program, Environmental Justice Thriving Communities Grantmaking Program Ej Tcgm, Environmental Policy and Innovation Grants, Environmental and Climate Justice Community Change Grants Program, Financial Assistance

		for Community Support Activities to Address Environmental Justice Issues, Greenhouse Gas Reduction Fund Clean Communities Investment Accelerator, Greenhouse Gas Reduction Fund National Clean Investment Fund, Greenhouse Gas Reduction Fund Solar for all, Healthy Communities Grant Program, National Wetland Program Development Grants and Five Star Restoration Training Grant
**NSF filter field**	**Designation**	**Values**
division	Environmental health	Atmospheric and Geospace Sciences, Earth Sciences, Multidisciplinary Activities, MPS Multidisciplinary Activities, Social and Economic Sciences
	Non-environmental health	Astronomical Sciences; Behavioral and Cognitive Sciences; Biological Infrastructure; Chemical, Bioengineering, Environmental and Transport Systems; Chemistry; Civil, Mechanical and Manufacturing Innovation; Computer and Network Systems; Computing and Communication Foundations; Emerging Frontiers and Multidisciplinary Activities; Engineering Education and Centers; Environmental Biology; Equity for Excellence in STEM; Graduate Education; Information and Intelligent Systems; Integrative Activities; Integrative Organismal Systems; International Science and Engineering; Materials Research; Mathematical Sciences; Molecular and Cellular Biosciences; Ocean Sciences; Office of Advanced Cyberinfrastructure; Physics; Polar Programs; Research on Learning in Formal and Informal Settings; Research, Innovation, Synergies, and Education; Technology, Innovation and Partnerships; Translational Impacts; Undergraduate Education
**NIH filter field**	**Designation**	**Values**
program_office	Environmental health	NIEHS
	Non-environmental health	FIC, NCATS, NCI, NEI, NHGRI, NHLBI, NIA, NIAAA, NIAID, NIAMS, NIBIB, NICHD, NIDA, NIDCD, NIDCR, NIDDK, NIGMS, NIMH, NIMHD, NINDS, NINR, NLM, OD
